# Extended-Release Trazodone in Comorbid Major Depressive and Sedative–Hypnotic/Anxiolytic Use Disorders: A Real-World Preliminary Evaluation of Multidimensional Outcomes

**DOI:** 10.3390/jcm15176874

**Published:** 2026-09-04

**Authors:** Marco Di Nicola, Maria Pepe, Francesca Tarantino, Ilaria Marcelli, Erica Bella, Lorenzo Bonomo, Raffaella Franza, Gabriele Sani

**Affiliations:** 1 Department of Neuroscience, Section of Psychiatry, Università Cattolica del Sacro Cuore, 00168 Rome, Italy; marco.dinicola@policlinicogemelli.it (M.D.N.);; 2 Department of Neurosciences, Head-Neck and Chest, Section of Psychiatry, Fondazione Policlinico Universitario Agostino Gemelli IRCCS, 00168 Rome, Italy; 3 Department of Anesthesiology and Intensive Care Medicine, Università Cattolica del Sacro Cuore, 00168 Rome, Italy; 4 Service of Clinical Psychology, Fondazione Policlinico Universitario Agostino Gemelli IRCCS, 00168 Rome, Italy

**Keywords:** dual diagnosis, comorbidity, antidepressants, personalized treatment, recovery

## Abstract

**Objectives**: Major depressive disorder (MDD) frequently co-occurs with substance use disorders, resulting in greater clinical severity and poorer outcomes. Sedative–hypnotic/anxiolytic agents (SHA) are commonly used to manage anxiety and insomnia, also in MDD. However, their long-term intake might lead to misuse and physical and cognitive complications. Trazodone, an antidepressant with sedative and anxiolytic properties, has been proposed as an option in SHA detoxification, but data on patients with comorbid MDD and SHA use disorders (MDD+SHA-UD) are limited. This study retrospectively evaluated the effects of a three-month treatment with extended-release trazodone in this population. **Methods**: Seventy-four outpatients with MDD+SHA-UD treated with extended-release trazodone were evaluated at baseline and after one and three months. Depressive symptoms were assessed using the Hamilton Depression Rating Scale. Secondary outcomes included anxiety (Hamilton Anxiety Rating Scale), sleep disturbances (Pittsburgh Sleep Quality Index), physical symptoms (Hamilton Depression–Anxiety subscales, 36-Item Short-Form Health Survey), cognitive functioning (Perceived Deficits Questionnaire–Depression, 5-item), and quality of life (World Health Organization-Five Well-Being Index). **Results**: Trazodone was associated with a significant reduction in depressive symptoms (*p* < 0.001), with 47.3% of the initial sample achieving remission at endpoint. Improvements were also observed in measures of anxiety, physical symptoms, subjective sleep quality, perceived cognitive functioning, and quality of life (all *p* < 0.001). Side effects were mild (25.7% at one month) and declined over time. **Conclusions**: In this uncontrolled preliminary study, extended-release trazodone was associated with significant improvements across multiple symptom domains in MDD+SHA-UD, warranting controlled investigation.

## 1. Introduction

Major depressive disorder (MDD) is among the most prevalent and disabling psychiatric conditions worldwide, affecting over 280 million individuals and accounting for a substantial healthcare, economic, and social burden [1]. MDD is primarily characterized by persistent low mood and anhedonia, but it also encompasses symptoms extending beyond the emotional domain [2]. These include cognitive dysfunction, somatic complaints such as fatigue and pain, psychomotor retardation, anxiety, and insomnia, each contributing to a worsened prognosis and functional impairment [2]. Such additional dimensions are frequently targeted with adjunctive pharmacological treatments, particularly sedative–hypnotic and anxiolytic agents (SHA), including benzodiazepines and Z-drugs. These compounds are widely prescribed for their short-term efficacy on anxiety and insomnia [3]. Although guidelines recommend limiting their use to the short term, real-world data show widespread chronic exposure [4]. Prolonged use has been linked to several adverse consequences including tolerance, cognitive impairment, paradoxical mood effects and, most notably, the development of dependence [5]. In recent years, concerns have grown also regarding the non-medical use of SHA, including intake without prescription, at higher-than-prescribed doses, or for recreational purposes [6], which is alarming because of the frequent association with polysubstance consumption and subsequent heightened risk of overdose, emergency visits, and hospitalization [7,8]. This phenomenon has been increasingly documented, especially among adolescents, women, and individuals with psychiatric diseases [9].

The comorbidity between MDD and substance use disorders (SUDs) is well established [10], but specific evidence on MDD in combination with SHA use disorder (SHA-UD) remains limited. Available studies indicate that individuals with SHA-UD are at higher risk of presenting MDD and, conversely, that SHA use is frequent in patients with more severe or treatment-resistant depressive symptoms, particularly when insomnia co-occurs [11,12]. This comorbidity complicates treatment, increases relapse risk, and is associated with reduced quality of life and higher healthcare utilization [13]. Nevertheless, no consensus guidelines exist for the pharmacological management of patients with MDD and SHA-UD. Interventions capable of addressing multiple symptom domains—such as depression, anxiety, sleep disturbance, somatic complaints, and cognitive dysfunction—while minimizing the risk of abuse are needed.

Trazodone, a serotonin antagonist and reuptake inhibitor (SARI) approved for the treatment of MDD in adults, may represent a promising candidate. Its pharmacological profile, including 5-HT2A antagonism, serotonin reuptake inhibition, α1-adrenergic blockade, and antihistaminergic effects, underlies its antidepressant, anxiolytic, and hypnotic properties [14]. The extended-release formulation provides smoother plasma levels and improved tolerability, potentially enhancing adherence [15]. Beyond its antidepressant efficacy, trazodone has shown some benefits for insomnia, anxiety, somatic and cognitive symptoms, and quality of life [16,17,18]. With low abuse liability, it has also been proposed as an option in patients with comorbid alcohol use disorder [19], and preliminary studies suggest its potential role in benzodiazepine detoxification [20].

On this basis, the present study retrospectively evaluated the effects of a three-month treatment with extended-release trazodone in outpatients with comorbid MDD and SHA-UD. Depressive symptoms were considered the primary outcome, while secondary analyses explored anxiety, physical symptoms, sleep quality, cognitive functioning, and overall quality of life, with the goal of providing preliminary evidence in an under-investigated clinical population.

## 2. Materials and Methods

### 2.1. Participants and Procedure

Outpatients who had consecutively referred to the “Centro Psichiatrico Integrato per la ricerca, la prevenzione e la cura delle Dipendenze” (CePID) at the Department of Psychiatry of Fondazione Policlinico Universitario “Agostino Gemelli” IRCCS in Rome, between January 2019 and January 2022, with a primary diagnosis of MDD comorbid with SHA-UD according to DSM-5 criteria were retrospectively screened for inclusion. Diagnoses had been confirmed through the Italian version of the Structured Clinical Interview for DSM-5 Disorders Clinician Version (SCID-5-CV). Subjects were considered eligible if they had at least moderate depression, as rated by a Hamilton Depression Rating Scale (HDRS) total score ≥ 14, and if they had been abstinent from SHAs for a minimum of 4 weeks before starting pharmacological treatment. Further inclusion criteria were age 18 to 65 years and fluency in spoken and written Italian. Exclusion criteria comprised other current substance use disorder, except for nicotine dependence; major medical disorders; and all conditions that could impair psychometric assessment, such as active psychotic features, organic brain syndromes, neurocognitive disorders or cognitive decline based on a Mini-Mental State Examination (MMSE) score < 26 [21]. Severity of the sedative, hypnotic and anxiolytic use disorder was characterized by the DSM-5 severity specifier and the number of criteria met, by the duration of the disorder, by the frequency and pattern of use, and by the dose taken on days of use before tapering, converted into diazepam equivalents according to the Ashton equivalence table [22] and summed across compounds in patients using more than one.

Before study inclusion, all patients had undergone gradual tapering of SHA until complete discontinuation, conducted on an outpatient basis according to a schedule of approximately 25% dose reduction per week, individualized according to the type and dose of the compound and to patient tolerability; the exact duration of tapering was not systematically recorded. Eligibility further required maintenance of complete abstinence for at least four weeks after discontinuation and before trazodone initiation, verified through clinical interview, self-report using a Timeline Followback calendar, and urine toxicology screening. Trazodone was therefore initiated in the post-withdrawal phase and was not used as an adjunct to detoxification; the withdrawal period preceded the observation window in its entirety, and standardized withdrawal severity scales were not administered.

Patients were prescribed extended-release trazodone (Angelini Pharma S.p.A., Rome, Italy) either prolonged-release (PR) 75–150 mg tablets or once-a-day (OAD) 150–300 mg formulations, at a flexible dose of 150–300 mg/day. The two formulations were pooled for analysis, as both deliver extended-release trazodone within the same daily dose range and the study was not designed to compare them. The initial dose and subsequent titration were individualized according to clinical presentation, response and tolerability, without a predefined titration schedule. Treatment adherence was monitored at each visit through clinical evaluation and patient self-report.

Concurrent use of other psychotropic medications was permitted, provided that regimens and dosages remained unchanged throughout the observation period; patients receiving antidepressants other than trazodone were excluded.

Patients also received a structured group psychotherapeutic intervention as part of the standard multidisciplinary treatment provided at the study center. Its content focused on psychoeducation about substance dependence, relapse prevention and management of high-risk situations, motivational enhancement, and systematic monitoring of craving and residual withdrawal symptoms. Group sessions were held weekly, lasted approximately 60–90 min, and were led by a clinical psychologist with specific expertise in addiction. The intervention had been initiated during the tapering phase, before study entry. The program was the same for all participants and continued unchanged throughout the three-month observation period.

Subjects who relapsed or discontinued the therapeutic-rehabilitation program were considered dropouts and no further assessments were collected after discontinuation. All available data from these participants—baseline and one-month assessments—were retained in the analyses.

Patients provided written informed consent for the use of their de-identified data for scientific purposes before enrollment, without receiving any form of compensation. The study protocol was conducted in accordance with the Good Clinical Practice guidelines and the Declaration of Helsinki (1964) and subsequent revisions and was approved by the Ethics Committee of the Fondazione Policlinico Universitario “A. Gemelli” IRCCS, Università Cattolica del Sacro Cuore, Rome (Italy) on 15 December 2022 (with the protocol number 5406).

### 2.2. Procedures and Assessment

Data were obtained from measurements and assessments regularly performed at baseline and after 1 and 3 months of treatment (endpoint) as part of routine clinical practice. Baseline data were collected at trazodone initiation. Depressive symptoms were evaluated using the 17-item HDRS. Patients were considered responders if they obtained at least a 50% reduction from baseline at endpoint, and remitters if the endpoint HDRS score was ≤7. For the categorical analysis, response and remission were treated as mutually exclusive categories: patients meeting remission criteria were classified as remitters; patients achieving a reduction of at least 50% without reaching an HDRS score ≤7 were classified as responders; the remaining completers were classified as non-responders. Exact numerators and denominators are reported for both the intention-to-treat and the completer samples.

Anxiety symptoms were assessed with the Hamilton Anxiety Rating Scale (HARS).

Physical symptomatology was evaluated with HDRS and HARS “physical” subscales and the “physical component score” (PCS) of the Short Form-36 Health Survey Questionnaire (SF-36). Specifically, physical features of depression were explored through items 4, 5, 6 (early, middle, late insomnia), 11 (somatic anxiety), 12 (gastrointestinal), 13 (general somatic, including muscular pain, headache, and lack of energy), 14 (genital—both loss of libido and menstrual disturbances), and 16 (loss of weight) of HDRS, and 7 (muscular pain), 8 (sensory), 9 (cardiovascular), 10 (respiratory), 11 (gastrointestinal), 12 (genitourinary) and 13 (autonomic) of HARS [2]. These item sets were defined *a priori* on the basis of item content, following the somatic clusters previously adopted by our and other groups [2,23] and according to the factorial solutions most commonly reported for the HDRS and the HARS physical subscales [23].

The SF-36 is a 36-item self-report survey of patients’ health consisting of eight sections, each ranging from 0 (worst health) to 100 (best health): bodily pain (BP); general health perceptions (GHPs); mental health (MH); physical functioning (PF); role emotional, i.e., limitation due to emotional problems (RE); role physical, i.e., limitation due to physical health problems (RP); social functioning (SF); and vitality (VT). Subdomain scores were aggregated into the PCS using the standard norm-based algorithm. Standardization was performed using the Italian normative data of the SF-36 [24], with the standard scoring coefficients of the original algorithm [25]. Higher scores indicate less physical disability; consistent with the norm-based metric, a score below 50 denotes physical functioning below the population average [26].

Additional outcome measures were evaluated at the same timepoints. Sleep disturbances were investigated by the self-rated Pittsburgh Sleep Quality Index (PSQI), using the cut-off total score of 5 to differentiate “good” from “bad” sleepers. Subjective cognitive functioning was assessed with the patient-rated Perceived Deficits Questionnaire-Depression, 5 items (PDQ-D5), with higher scores (range 0–20) indicating greater perceived impairment in attention/concentration, planning/organization, and retrospective and prospective memory. The World Health Organization-Five Well-Being Index (WHO-5) was also administered. Each of its five items is rated from 0 to 5, giving a raw score between 0 and 25, which is conventionally multiplied by 4 to yield a percentage score ranging from 0 (worst imaginable well-being) to 100 (best imaginable well-being). A percentage score of 28 or below has been proposed as a screening cut-off for DSM-IV major depression; a change of 10 points on the percentage scale is regarded as clinically relevant [27]. Raw scores are reported throughout the manuscript, and the corresponding percentage values are given where relevant.

The safety and tolerability of trazodone were assessed by physical examination; baseline and endpoint measurement of body weight, height and Body Mass Index (BMI); routine laboratory and instrumental clinical tests (e.g., electrocardiograms with corrected QT interval calculation); and patients’ reports of any adverse events. All patients had complete baseline laboratory and electrocardiographic data, and follow-up assessments were performed as part of routine clinical care. Pre–post-comparisons are based on complete cases, and the number of patients contributing to each comparison is reported. Laboratory values were considered abnormal when above the upper limit of normal of the local laboratory, and aminotransferase elevations exceeding three times that limit were regarded as clinically relevant, in line with the criteria of the FDA guidance on drug-induced liver injury [28]. Thresholds of clinical concern were defined according to ICH E14 [29], using absolute QTc values above 450, 480 and 500 ms and increases from baseline exceeding 30 and 60 ms. Adverse events were recorded as spontaneously reported by patients and as assessed by the treating clinician at each visit.

### 2.3. Statistical Analysis

Descriptive data are summarized as number and percentage (%) or mean ± standard deviation (M ± SD) for dichotomous and continuous variables, respectively. Changes in each outcome were analyzed using linear mixed models with a random intercept for participant; time (baseline, one month, three months) entered as a fixed factor; and age, sex, illness duration, and concomitant psychotropic medication included as covariates. Models were estimated by restricted maximum likelihood with Satterthwaite degrees of freedom. This approach uses all available observations without case deletion. Estimates are reported with 95% Wald confidence intervals. Standardized mean differences were obtained by dividing the model-estimated change from baseline by the observed baseline standard deviation, with confidence intervals derived by the same standardization; they therefore express the precision of the estimated change on the standardized scale. Semi-partial R^2^ (R^2^β) for the effect of time was computed to express the magnitude of the time effect as a proportion of variance explained. Pairwise comparisons between timepoints were Bonferroni-corrected within each outcome, and the Benjamini–Hochberg procedure was applied to the seven omnibus tests of the effect of time across the secondary outcomes. Model assumptions were checked for each model by inspection of Q–Q plots and histograms of the conditional residuals, residual-versus-fitted plots, and histograms of the estimated random intercepts and by the Shapiro–Wilk test on the conditional residuals. Diagnostics are reported in Supplementary Table S1.

No *a priori* sample size calculation was performed, and all patients meeting the inclusion criteria and treated during the observation period were included, so the sample size was determined by the size of the eligible cohort.

The primary and anxiety outcome models were also re-estimated on non-somatic scores, obtained by removing the physical items from the respective total scores, to establish whether the improvement observed in the total scores was attributable to their somatic and sleep components.

Similarly, an exploratory item-level analysis of the individual HDRS and HARS physical items was performed with Wilcoxon signed-rank tests comparing baseline and endpoint, with Benjamini–Hochberg correction across the items, to verify whether the change in the subscale totals was distributed across the individual symptoms or driven by a subset of them.

In a separate set of models, baseline trazodone dose (mg/day) was included as a further continuous covariate.

To explore the potential confounding contribution of concomitant psychotropic treatment, exploratory models including a time × concomitant medication interaction were fitted for each outcome, with the simple effects of time estimated within each subgroup. These analyses were not planned *a priori* and are reported without correction for multiplicity.

To assess the robustness of the primary outcome to potentially informative attrition, a delta-adjusted sensitivity analysis was performed. For each of the twelve discontinuers, the three-month HDRS value was imputed as their last observed score penalized by an increment δ, with δ increased progressively from 1 to 8 points to represent departures from the missing-at-random assumption of increasing severity. The mixed model described above was re-estimated across this range to identify the tipping point at which the effect of time would cease to be significant. In addition, a boundary scenario was retained in which missing three-month values were imputed under a conservative deterministic assumption: baseline values were carried forward for the patients who relapsed into sedative–hypnotic use, assuming no sustained treatment benefit, and one-month values were carried forward for the remaining discontinuers, assuming that the improvement observed at one month was retained but that no further benefit accrued. This scenario is deterministic and does not propagate imputation uncertainty; it is reported for all outcomes as an extreme boundary condition.

For clinical parameters (i.e., liver function tests and electrocardiographic values), changes between baseline and endpoint were tested using paired samples *t*-tests or Wilcoxon Signed Ranks Tests, according to data distribution. Specifically, aminotransferases showed non-normal distributions and were analyzed with the Wilcoxon signed-rank test, whereas gamma-glutamyl transferase and corrected QT interval were normally distributed and analyzed with paired *t*-tests. A significance level of 0.05 was used for each test. All analyses were conducted using IBM SPSS Statistics for Windows, v. 28.0 (IBM Corp., Armonk, NY, USA).

## 3. Results

Eighty-nine patients were screened for enrollment. After excluding those who did not provide informed consent (*n* = 10) and participants with incomplete chart data (*n* = 5), 74 Caucasian subjects with MDD+SHA-UD were included. Sociodemographic, clinical, and psychometric characteristics at baseline are summarized in Table 1.

Thirty-two patients (43.2%) presented an additional psychiatric diagnosis (specifically anxiety disorders in 25.7%, eating disorders in 8.1%, obsessive–compulsive disorder in 4.1%, pathological gambling in 2.7%, and neurodevelopmental disorders in 2.7%), and 27.0% reported lifetime substance abuse. All patients met DSM-5 criteria for moderate (*n* = 32, 43.2%) or severe (*n* = 42, 56.8%) sedative-hypnotic/anxiolytic use disorder. Use was daily in 33 patients (44.6%), four to six times per week in 26 (35.1%), and one to three times per week in 15 (20.3%). Nineteen patients (25.7%) used more than one compound concurrently; in these cases, the main compound was identified as the one taken at the highest diazepam-equivalent dose. The main compound was alprazolam in 23.0% of patients, lormetazepam in 18.9%, zolpidem in 16.2%, lorazepam in 14.9% and clonazepam in 9.5%, with the remaining patients (17.6%) using other agents. Thirty patients (40.5%) were prescribed the prolonged-release formulation, while 44 (59.5%) assumed the once-a-day formulation, within a flexible dose range of 150–300 mg/day. Concomitant psychopharmacological treatment was recorded in 53 patients (71.6%); among them, 37 (69.8%) were receiving mood stabilizers or anticonvulsants and 22 (41.5%) antipsychotics. The two categories are not mutually exclusive.

At the endpoint, data were available for 62 subjects, with all dropouts occurring after the first month of treatment (dropout rate: 16.2%, *n* = 12). According to available clinical records, none of these cases reflected pharmacological discontinuation due to inefficacy or adverse effects; discontinuation was attributed to non-clinical reasons in 7 patients (58.3%) and to relapse into SHA use in 5 patients (41.7%). Overall, 210 of the 222 scheduled assessments were attended (94.6%).

Results for both primary and secondary outcome measures are reported in Table 2.

Depressive symptom severity decreased progressively over the three-month period, with significant reductions at one month that continued through three months. The effect of time was significant, F(2, 139) = 344.2, *p* < 0.001, R^2^β = 0.832. Estimated marginal mean HDRS scores fell to 12.66 (11.84–13.48) at one month and 6.44 (5.75–7.13) at three months. All pairwise comparisons remained significant after Bonferroni correction: baseline to one month (adjusted mean difference 4.87, t = 15.4, *p* < 0.001), baseline to three months (11.1, t = 25.8, *p* < 0.001), and one to three months (6.20, t = 11.5, *p* < 0.001). No effects of age, sex, illness duration, or concomitant psychotropic medication were detected (all *p* ≥ 0.05). On an intention-to-treat basis (*n* = 74), at endpoint, 35 of 74 patients (47.3%) met remission criteria, 17 of 74 (23.0%) achieved response without remission, and 10 of 74 (13.5%) were non-responders; the remaining 12 of 74 (16.2%) discontinued before the endpoint. Among the 62 patients who completed the three-month assessment, the corresponding rates were 35 of 62 (56.5%) for remission, 17 of 62 (27.4%) for response without remission, and 10 of 62 (16.1%) for non-response.

When baseline trazodone dose was added to the models, it was not significantly associated with any outcome (all *p* ≥ 0.353), and the effect of time remained significant throughout, with essentially unchanged estimated marginal means.

Anxiety severity declined over the three months. Estimated marginal HARS scores decreased to 16.8 (16.21–17.39) at one month and 10.9 (10.18–11.62) at three months, with a significant effect of time, F(2, 138) = 258.0, *p* < 0.001, R^2^β = 0.789. All pairwise contrasts remained significant after Bonferroni correction: baseline to one month (adjusted mean difference 4.26, *p* < 0.001), baseline to three months (10.20, *p* < 0.001), and one to three months (5.94, *p* < 0.001). No effects of age, sex, illness duration, and concomitant psychotropic medication were detected (all *p* ≥ 0.05).

Physical symptoms improved consistently across three scales. On the HDRS physical subscale, estimated marginal scores reduced to 4.43 (4.02–4.84) at one month and 2.08 (1.64–2.52) at three months (F(2, 138) = 200.7, *p* < 0.001, R^2^β = 0.744). The HARS physical subscale declined to 5.45 (5.01–5.91) and 3.81 (3.32–4.29) (F(2, 137) = 120.9, *p* < 0.001, R^2^β = 0.638). Physical health-related quality of life on the SF-36 PCS increased correspondingly, to 40.6 (39.4–41.8) at one month and 49.8 (48.7–50.9) at three months (F(2, 134) = 243.5, *p* < 0.001, R^2^β = 0.784), approaching the population normative value of 50 by endpoint. More specifically, 36 patients reached an SF-36 PCS score ≥ 50 at endpoint, indicating good physical functioning—58.1% of completers and 48.6% of the enrolled sample.

All pairwise contrasts were significant after Bonferroni correction (all *p* < 0.001). A sex difference was confined to the HDRS physical subscale, on which women scored higher than men (adjusted difference 1.05 points, 95% CI 0.42–1.68, *p* = 0.007); 12 of the 27 women (44.4%) were aged between 45 and 55 years. No sex effect was present on the HARS physical subscale (*p* = 0.27) or the SF-36 physical component (*p* = 0.37). A time × sex interaction on the HDRS physical subscale was non-significant (F(2, 131) = 0.43, *p* = 0.76), suggesting that the female elevation was stable across assessments, with both sexes improving in parallel (women vs. men: 7.27 vs. 5.99 at baseline, 2.49 vs. 1.62 at three months).

An exploratory item-level analysis showed significant improvement from baseline to three months in every individual physical item of both HDRS and HARS after Benjamini–Hochberg correction (all adjusted *p* ≤ 0.003), indicating that the change in the subscale totals was distributed across the symptoms they aggregate rather than driven by a subset of items (Supplementary Table S2).

When the analyses were repeated on non-somatic scores, obtained by removing the physical items from the respective totals, the effect of time remained large and significant for both instruments (HDRS: F(2, 136) = 144.5, *p* < 0.001, R^2^β = 0.680; HARS: F(2, 138) = 110.1, *p* < 0.001, R^2^β = 0.615). The adjusted reductions from baseline to endpoint were 6.52 points on the non-somatic HDRS (95% CI 5.76 to 7.28; d = 2.61) and 6.33 points on the non-somatic HARS (95% CI 5.48 to 7.18; d = 2.32), corresponding to 59% and 62% of the reductions observed on the respective total scores.

Improvements extended to sleep, cognition, and subjective well-being, each showing a significant effect of time (all *p* < 0.001) with no significant effect of age, sex, illness duration, or concomitant medication (all *p* > 0.10). Self-perceived sleep quality (PSQI) improved from baseline values indicating clearly disturbed sleep to a mean score of 10.6 (9.63–11.6) at one month and 5.14 (4.1–6.2) at three months, approaching the conventional threshold for good sleep quality by endpoint (F(2, 136) = 158.5, R^2^β = 0.700). Specifically, at three months, 45 patients were classified as good sleepers (PSQI scores ≤5)—72.6% of completers and 60.8% of the enrolled sample. Perceived cognitive deficits (PDQ-D5) declined to 8.88 (8.28–9.49) and 4.82 (4.16–5.47) over the same intervals (F(2, 134) = 179.5, R^2^β = 0.728). Subjective well-being (WHO-5), scored in the opposite direction, rose from baseline mean values indicative of poor well-being to a mean score of 9.23 (8.53–9.93) at one month and 15.73 (14.98–16.48) at three months (F(2, 134) = 384.3, R^2^β = 0.852). On the percentage scale, the estimated marginal means correspond to a rise from 26.2% at baseline to 62.9% at endpoint; the adjusted increase of 36.8 percentage points is well beyond the 10-point change regarded as clinically relevant. For all three measures, every pairwise contrast was still significant after Bonferroni correction (all *p* < 0.001; Table 2).

Across all secondary outcomes, the effect of time remained significant for every measure after Benjamini–Hochberg correction (all adjusted *p* < 0.001).

Effect sizes are reported for all outcomes at endpoint (standardized mean differences ranging from 1.82 to 4.03; Table 2), and the effect of time accounted for a substantial proportion of variance (semi-partial R^2^β 0.638 to 0.852).

In the delta-adjusted sensitivity analysis, the effect of time on HDRS scores remained significant across the entire range examined (all *p* < 0.001), including the most extreme scenario, in which all discontinuers were assumed to have deteriorated by 8 points beyond their last observed score. Since in this subgroup the mean difference between baseline and one-month scores was 6.8 points, increments above this value bring the mean imputed endpoint score above the mean baseline score of the subgroup, so the upper end of the range tested is, on average, more severe than the assumption of carrying baseline values forward. No tipping point was identified within a clinically plausible range of departures from the missing-at-random assumption. The deterministic boundary scenario, reported for all outcomes, likewise confirmed that every measure retained significant improvement over time (F values ranging from 95.4 to 199.7, all *p* < 0.001), with effect estimates expectedly attenuated but directionally unchanged (Supplementary Table S3).

In exploratory models including a time × concomitant psychotropic medication interaction, the interaction term was non-significant for every outcome except the HDRS total score, where it reached nominal significance (F(2, 137) = 4.18, *p* = 0.018) but did not survive correction for multiplicity across the eight tests. The effect of time was significant within both subgroups for all outcomes, with changes of comparable magnitude, and Bonferroni-corrected pairwise comparisons between subgroups at each assessment point were non-significant throughout (all *p* ≥ 0.667; Supplementary Tables S4 and S5).

Regarding laboratory parameters, the number of patients displaying baseline values above the upper limit of normal was 22 of 74 (29.7%) for AST, 9 of 74 (12.2%) for ALT, and 24 of 74 (32.4%) for GGT. No patient exceeded three times the upper limit for AST or ALT. At baseline, 1 of 74 patients (1.4%) showed a QTc value above 450 ms, and none above 480 or 500 ms.

At endpoint, patients displayed significant reductions in liver function tests (AST: 40.1 ± 16.7 IU/L vs. 29.3 ± 8.86 IU/L, W = 495, *p* < 0.001; ALT: 29.6 ± 17.3 IU/L vs. 20.1 ± 9.59 IU/L, W = 458, *p* < 0.001; GGT: 48.5 ± 38.5 IU/L vs. 30.2 ± 34.3 IU/L, t = 4.50, *p* < 0.001; *n* = 55). The number of subjects displaying endpoint values above the upper limit of normal was 7 of 55 (12.7%) for AST, 0 of 55 for ALT, and 8 of 55 (14.5%) for GGT. No patient exceeded three times the upper limit for AST or ALT. No significant baseline-endpoint change occurred in the QTc interval (402.7 ± 19.4 ms vs. 397.5 ± 14.7 ms; mean change −5.2 ms; t = 1.88, *p* = 0.07; *n* = 54). At endpoint, no patient showed a QTc value above 450, 480 or 500 ms, and no increase from baseline exceeded 30 ms; consequently, none exceeded 60 ms.

Finally, adverse events were reported within the first month of treatment by 19 of the 74 patients (25.7%): drowsiness or dizziness in 12 (16.2%), dry mouth in 4 (5.4%), and headache in 3 (4.1%). All events were rated as mild, and none led to dose reduction or treatment discontinuation. At endpoint, 6 of the 62 patients still in treatment (9.7%) reported persistent, albeit mild, drowsiness or dizziness.

## 4. Discussion

In this real-world, retrospective study, we observed that patients diagnosed with comorbid major depressive and sedative–hypnotic/anxiolytic use disorders who were treated with extended-release trazodone reported significant improvements across multiple psychopathological dimensions. Antidepressant response was already evident at one month and continued to increase throughout the three-month follow-up. By endpoint, the majority of patients had experienced clinical improvement, and remission was observed in nearly half of the enrolled sample. These findings align with prior evidence supporting the antidepressant efficacy of trazodone in MDD populations [30] while extending the available knowledge to a more complex population with dual diagnoses. Moreover, improvements also encompassed anxiety, subjectively reported sleep quality, physical symptomatology, self-perceived cognitive difficulties, and overall quality of life, alongside a good safety/tolerability profile. These concurrent improvements suggest a broad-spectrum action of trazodone in this clinical context; however, it should be noted that the uncontrolled design means they cannot be completely disentangled from other factors, including ongoing abstinence and concomitant interventions.

The magnitude of these estimates warrants some considerations. In an uncontrolled study design, the effect of time is likely to reflect the combined contribution of multiple processes operating throughout the observation period—including sustained abstinence, patient expectancy effects, ongoing psychosocial support and additional pharmacotherapy—and therefore cannot be interpreted as a direct measure of the efficacy of trazodone. Moreover, two characteristics of the sample may have contributed to the magnitude of both the variance explained and the standardized mean differences, compared with the values observed in more heterogeneous cohorts. First, the inclusion criterion of a baseline HDRS total score of at least 14 restricted the range of baseline symptom severity. Second, recruitment from a single center implementing a standardized rehabilitation program likely resulted in a relatively homogeneous sample. Since the standardized mean differences were computed on the observed baseline standard deviations, which were correspondingly narrow, they are not directly comparable with effect sizes derived from more heterogeneous trial populations. Accordingly, these values should be interpreted primarily as descriptors of the observed trajectories rather than as evidence of a specific pharmacological effect.

The high prevalence of benzodiazepine and Z-drug prescriptions as short-term adjuncts for anxiety and insomnia among depressed patients is well documented [13]. In Italy, benzodiazepines are among the most prescribed psychotropic medications in outpatient care, with an estimated consumption of around 40 defined daily doses per 1000 inhabitants per day, and over 60% of prescriptions extending beyond three months, especially in women and the elderly, whereas similar though less pronounced patterns are observed for Z-drugs [31]. Clinical studies further indicate that SHAs are frequently co-prescribed in MDD patients already on antidepressants, and that such co-treatment tends to persist in the long term [3,32]. This widespread and chronic exposure is concerning, given that approximately 17% of benzodiazepine users and 9% of Z-drug users report misuse, and 12–14% of past-year misusers meet diagnostic criteria for SHA-UD [33].

The association between MDD and SHA-UD is supported by epidemiological evidence indicating higher odds of dual diagnosis [11,12,13]. Indeed, SHA misuse frequently arises from self-medication motives, such as attempts to alleviate anxiety, stress, or sleep disturbances [34,35]. An early onset of depression has been identified as a strong predictor of subsequent SHA-UD [11], and treatment-resistant depression is a significant risk factor for later substance use disorders, particularly SHA-UD [36]. Conversely, long-term benzodiazepine exposure may alter GABAergic and monoaminergic homeostasis, worsening mood instability and precipitating withdrawal-related depressive episodes [37]. Long-term SHA exposure has also been associated with an increased likelihood of developing mood disorders, with greater severity of depression itself and functional impairment [5,38]. Together, these findings support a bidirectional relationship, whereby mood instability promotes misuse, and misuse, in turn, worsens mood and functional outcomes [32,39].

Addressing adjunctive symptoms such as anxiety, insomnia, and physical features, without adding to the risk of dependence, represents a critical therapeutic need. Sleep disturbances and anxiety are also the primary reasons for SHA prescription in MDD and—especially if persistent—can contribute to treatment resistance, greater clinical severity and increased relapse risk in both mood and addiction disorders. Somatic symptoms such as pain, fatigue, gastrointestinal complaints, and muscle tension are common in MDD [2] and in SHA misuse, related to both intoxication and withdrawal, and have been associated with poor prognosis [5]. In this context, trazodone may represent a suitable intervention. Preliminary evidence has suggested its utility in benzodiazepine detoxification, likely due to its efficacy on prolonged withdrawal symptoms such as anxiety, insomnia and somatic complaints [40]. In line with prior reports [18,41], in our sample, the extended-release formulation was associated with significant reductions in anxiety and improvements in sleep quality, supporting the potential of trazodone to complement personalized treatment and current recommendations of gradual tapering for SHA-UD treatment [42,43]. Here, physical symptomatology also improved significantly both in terms of HDRS and HARS subscales and in the SF-36 scores. These findings are consistent with prior work indicating beneficial effects of trazodone on somatic symptoms in depressive syndromes and other conditions such as fibromyalgia and neuropathic pain [44,45]. A recent study also reported the effectiveness of trazodone vs. SSRIs in improving pain/discomfort symptoms and health-related quality of life [30]. Notably, sex differences have been documented, with women often presenting more prominent somatic depressive symptoms [13], which may explain our observation of higher mean scores in such domains for female participants. However, this difference was confined to the HDRS physical subscale, which captures symptoms potentially overlapping with the menopausal transition, whereas no sex differences emerged on the HARS physical subscale or the SF-36 physical component. Given the age distribution of the female participants, an unmeasured contribution of perimenopausal symptoms cannot be excluded but warrants further investigation [46], as hormonal status was not assessed. The HDRS and HARS physical subscales are subsets of their respective total scores, so the two measures are not independent and their concurrent improvement should not be read as evidence of two entirely separate effects. In addition, HDRS items 4–6 index insomnia and therefore overlap in content with the PSQI, so the parallel improvement of these two measures could be, at least in part, attributable to shared item content as well. When the analyses were repeated after removing the physical items from the total scores, however, the effect of time on the residual non-somatic scores remained large for both instruments, indicating that the improvement in mood and psychic anxiety is not reducible to the somatic and sleep domains. Furthermore, the HDRS and HARS physical subscales aggregate symptoms of heterogeneous nature—insomnia, gastrointestinal, genitourinary, autonomic and pain-related complaints—which may not respond uniformly to treatment. The subscale totals should therefore be interpreted as global indices of somatic burden.

Importantly, although trazodone may relieve the anxiety, insomnia, and somatic symptoms accompanying prolonged SHA withdrawal, it does not prevent the potentially serious manifestations of benzodiazepine withdrawal, such as seizures. It should accordingly be regarded as an adjunct to, not a substitute for, appropriate gradual tapering under clinical supervision [42,43].

Cognitive dysfunction is increasingly recognized as a key residual symptom of depression contributing to functional impairment and relapse [47,48]. Benzodiazepines and Z-drugs are also known to negatively affect cognition, especially with long-term use [49]. In our study, patients reported significant improvements in perceived cognitive functioning, conceivably attributable to a combination of factors such as improvements in mood, sustained abstinence from SHA [49] and, possibly, trazodone’s pharmacological effects on dopaminergic pathways and neuroplasticity [17]. Future prospective studies with performance-based neurocognitive testing and longer follow-up should disentangle the relative contributions of abstinence and pharmacotherapy to cognitive recovery.

Beyond symptom domains, trazodone treatment was associated with improvements in subjective well-being and functioning, as reflected by significant increases in WHO-5 scores. Quality of life is often severely compromised in patients with dual diagnoses, reflecting the combined burden of depressive symptomatology, substance misuse, and social or occupational impairment [18,19,41]. Improving psychosocial functioning is now regarded as a central therapeutic target that complements symptomatic remission [47]. Therefore, our findings reinforce the potential of trazodone to contribute to broader recovery trajectories in this population.

The pharmacological profile of trazodone—serotonin reuptake inhibition, 5-HT2A antagonism, α1-adrenergic blockade, and antihistaminergic effects—might support improvements across multiple domains [14], thus providing a rationale for its effectiveness in such complex patients. The serotonergic modulation underpins its antidepressant properties, while α1-adrenergic antagonism contributes to reductions in somatic anxiety, restlessness, and autonomic symptoms. Histamine receptor antagonism facilitates sleep onset and maintenance, which may be particularly beneficial in SHA-UD patients, where insomnia often drives continued substance misuse [34,35]. Finally, emerging evidence suggests that trazodone exerts indirect dopaminergic modulation in the prefrontal cortex, promoting neuroplasticity and enhancing cognitive performance [17]. This mechanistic versatility supports the role of trazodone as a flexible tool for patients with comorbidities and multidimensional symptom profiles [16,19,47].

Safety and tolerability findings from this study were consistent with the established profile of trazodone, in which somnolence and sedation are the most frequent events, followed by dizziness, dry mouth and headache, whereas anticholinergic, sexual and metabolic events are comparatively uncommon [14,16,17]. The same ordering was observed in our cohort, where drowsiness or dizziness were the most frequently reported events, followed by dry mouth and headache, in keeping with the randomized trials of the same extended-release formulation [50,51]. Adverse events were, however, ascertained through spontaneous report and clinical inquiry rather than with a structured checklist, an approach that may yield lower event rates than systematic elicitation; the frequencies observed here are therefore not directly comparable with those reported in those trials and do not provide generalizable estimates of incidence, although the overall picture is one of good tolerability. Discontinuation is not subject to the same limitation: no patient in our cohort discontinued because of an adverse event, whereas adverse-event-related withdrawals were reported in both trials.

No worsening was observed in aminotransferase and gamma-glutamyl transferase levels, supporting the absence of a hepatotoxic signal over three months in this high-risk population. The overall reductions in liver function parameters occurred during a period of sustained abstinence from sedative–hypnotic agents; their determinants were beyond the aims of this study and cannot be established from the present data.

Trazodone treatment was not associated with electrocardiographic abnormalities, and at endpoint, no patient exceeded any of the absolute or change-from-baseline thresholds recommended for the identification of outliers. Since rare cases of QT prolongation have been reported with trazodone, and concomitant use with other QT-prolonging agents may increase the risk of ventricular arrhythmia [52], our findings are compatible with, but do not demonstrate, cardiac safety, and electrocardiographic monitoring remains advisable in patients with cardiac comorbidity, electrolyte disturbances, or concomitant use of QT-prolonging agents.

### Strengths and Limitations

To our knowledge, this is the first study to evaluate the effects of extended-release trazodone across multiple psychopathological dimensions in comorbid MDD+SHA-UD patients in a naturalistic clinical setting. The cohort was characterized with standardized indices of the severity of the preceding use disorder, and treatment was monitored with laboratory and electrocardiographic parameters alongside psychometric outcomes.

Several limitations must nonetheless be acknowledged. The design was retrospective, observational, monocentric and uncontrolled, which limits the extent to which the improvements observed can be specifically associated with extended-release trazodone. The trajectories may reflect, at least in part, symptomatic improvement following sustained abstinence, resolution of protracted withdrawal phenomena, non-specific benefits associated with participation in a structured rehabilitation program and regular clinical contact, and patient expectancy. Regression to the mean may also have contributed, since eligibility required a baseline HDRS total score of at least 14. Randomized controlled trials with an active comparator or a treatment-as-usual arm would be valuable to better delineate the specific contribution of extended-release trazodone to the observed improvements.

Outcomes were derived from clinician- or self-rated scales, and sleep, cognition and quality of life relied exclusively on subjective instruments; in patients who have recently discontinued sedative–hypnotics, such ratings may be particularly susceptible to expectancy effects and to the resolution of withdrawal-related distress. Future studies should incorporate objective indices, such as actigraphy or polysomnography for sleep, performance-based neuropsychological testing for cognition, and clinician-rated or performance-based measures of functioning.

Turning to treatment, trazodone dose was determined on clinical grounds rather than randomized, so any dose–response relationship observed in this cohort is open to confounding by indication and should be regarded as exploratory. Treatment adherence was assessed clinically, and standardized quantitative measures (e.g., pill count) were not performed. About 70% of the sample received concomitant psychopharmacotherapy (mainly mood stabilizers/anticonvulsants and antipsychotics); although these treatments were left unchanged throughout the observation period, their inclusion as covariates did not modify the results, and exploratory models including a time × medication interaction showed comparable trajectories in the two subgroups. However, their potential independent or synergistic effects on mood, anxiety, physical symptoms, sleep and cognition cannot be fully disentangled in an observational study of this size, and studies stratified by, or restricted to, defined medication regimens are therefore needed.

Although standardized indices of the severity of the SHA use disorder were available, withdrawal severity was not assessed prospectively and the duration of tapering was not systematically recorded, since discontinuation preceded study entry. We therefore cannot establish whether the intensity of the preceding withdrawal moderated the response to trazodone.

Finally, the sample size, while adequate for the within-subject longitudinal contrasts, limits the precision of the estimates of the between-subject terms—age, sex, illness duration, concomitant medication, and the time × sex interaction—and the follow-up period was short. These features limit the generalizability of the findings, which should be regarded as preliminary and as requiring confirmation in larger, controlled, prospective studies.

## 5. Conclusions

The prevalence of comorbid psychiatric and substance use disorders is steadily increasing, while pharmacological guidelines for their management remain limited. Current recommendations generally emphasize abstinence before initiating treatment, yet delaying interventions in patients with a high psychopathological burden may contribute to poorer outcomes. Moreover, individuals with active substance misuse are typically excluded from clinical trials, given that most protocols require prolonged abstinence, thereby leaving clinicians reliant on real-world evidence to guide treatment strategies in this population.

Therapeutic approaches specifically targeting patients with MDD and SHA-UD are scarce, despite the substantial psychopathological burden these patients usually present. Symptoms such as anxiety, insomnia, and somatic complaints often interact and amplify clinical severity, particularly during withdrawal. The clinical heterogeneity of this population further highlights the need for tailored interventions. Within this framework, extended-release trazodone deserves further investigation. In our sample, the three-month treatment period was accompanied by significant reductions in depressive, anxiety and somatic symptoms and by improvements in subjectively reported sleep, perceived cognition and quality of life, with good tolerability. Because the study was retrospective and uncontrolled, and because pharmacological treatment was delivered alongside sustained abstinence and a structured psychosocial program, these changes cannot be attributed specifically and solely to trazodone and should be regarded as preliminary, hypothesis-generating observations. However, the multidimensional improvements appear to be clinically relevant, as they address the key domains that often complicate the management of comorbid MDD and SHA-UD. Future prospective studies with larger cohorts and longer observation periods are warranted to confirm these preliminary results and to better define the role of trazodone in personalized treatment strategies for this under-investigated population.

## Figures and Tables

**Table 1:** Baseline sociodemographic and clinical characteristics of the sample.

*Characteristics (n, %, M ± SD)*	
**Overall**	74
*Sociodemographic*	
**Age** (years)	46.6 ± 10.9
**Gender**	
Female	27 (36.5)
Male	47 (63.5)
**Education level**	
Primary school	3 (4.1)
Middle school	18 (24.3)
High school	38 (51.3)
University	15 (20.3)
**Employment** (employed)	42 (56.7)
**Marital status** (married)	39 (52.7)
*Clinical*	
**BMI**	25.2 ± 4.45
**Medical comorbidities** (yes)	42 (56.7)
**Smoking** (yes)	43 (58.1)
**Age at onset** (years)	29.7 ± 12.8
**Duration of illness** (years)	16.4 ± 9.96
**SHA-UD duration (years)**	8.89 ± 5.41
**DSM-5 criteria met**	6.25 ± 1.79
**Diazepam equivalents (mg)**	40.5 ± 22.0
**Family history of psychiatric disorders** (yes)	47 (63.5)
**Trazodone dosage** (mg/day)	209 ± 64.7
*Psychometric*	
**HDRS**	17.3 ± 3.43
*Physical Symptoms*	6.36 ± 2.25
**HARS**	21.1 ± 2.53
*Physical Symptoms*	7.72 ± 1.75
**SF-36 PCS**	36.8 ± 4.77
**PDQ-D5**	11.4 ± 2.47
**PSQI**	13.0 ± 4.35
**WHO-5**	6.64 ± 2.30

**Abbreviations**. BMI, Body Mass Index; HARS, Hamilton Anxiety Rating Scale; HDRS, Hamilton Depression Rating Scale; M, mean; PDQ-D5, Perceived Deficits Questionnaire for Depression; PSQI, Pittsburgh Sleep Quality Index; SD, Standard Deviation; SF-36, 36-Item Short Form Survey (PCS, Physical Component Score); WHO-5, World Health Organization-Five Well-Being Index. *Note*: Psychometric assessment scores at baseline are reported as observed means. Diazepam equivalents on days of use were converted according to the Ashton equivalence table and summed across compounds in patients using more than one (range: 10–88 mg).

**Table 2:** Changes in primary and secondary outcome measures at different time-points (LMM).

	M ± SD	Change from Baseline, M (SE)	95% CI	*t*	*p*	d [95% CI]
**HDRS–Total**						
*1* m	12.4 ± 2.66	−4.87 (0.32)	−5.50, −4.24	−15.4	**<0.001**	−1.42 [−1.60, −1.24]
*3* m	6.27 ± 2.07	−11.10 (0.43)	−11.95, −10.25	−25.8	**<0.001**	−3.24 [−3.48, −2.99]
**HDRS–Physical**						
*1* m	4.19 ± 1.40	−2.17 (0.21)	−2.59, −1.75	−10.1	**<0.001**	−0.96 [−1.15, −0.78]
*3* m	1.77 ± 1.26	−4.52 (0.23)	−4.97, −4.07	−19.9	**<0.001**	−2.01 [−2.21, −1.81]
**HARS–Total**						
*1* m	16.7 ± 2.48	−4.26 (0.43)	−5.11, −3.41	−9.91	**<0.001**	−1.68 [−2.02, −1.35]
*3* m	10.8 ± 3.10	−10.20 (0.45)	−11.09, −9.31	−22.6	**<0.001**	−4.03 [−4.38, −3.68]
**HARS–Physical**						
*1* m	5.55 ± 1.94	−2.17 (0.23)	−2.62, −1.72	−9.43	**<0.001**	−1.24 [−1.50, −0.98]
*3* m	3.80 ± 1.61	−3.81 (0.25)	−4.30, −3.32	−15.3	**<0.001**	−2.18 [−2.46, −1.89]
**SF-36 PCS**						
*1* m	40.4 ± 4.50	+3.59 (0.56)	2.48, 4.70	6.41	**<0.001**	+0.75 [0.52, 0.98]
*3* m	50.0 ± 3.53	+12.90 (0.59)	11.73, 14.07	21.6	**<0.001**	+2.70 [2.46, 2.95]
**PDQ-D5**						
*1* m	8.81 ± 2.31	−2.56 (0.33)	−3.21, −1.91	−7.75	**<0.001**	−1.04 [−1.30, −0.77]
*3* m	4.77 ± 2.17	−6.63 (0.35)	−7.32, −5.94	−18.7	**<0.001**	−2.68 [−2.96, −2.40]
**PSQI**						
*1* m	10.5 ± 3.88	−2.47 (0.42)	−3.30, −1.64	−5.88	**<0.001**	−0.57 [−0.76, −0.38]
*3* m	4.93 ± 1.45	−7.93 (0.45)	−8.82, −7.04	−17.6	**<0.001**	−1.82 [−2.03, −1.62]
**WHO-5**						
*1* m	9.34 ± 2.46	+2.69 (0.31)	2.08, 3.30	8.52	**<0.001**	+1.17 [0.90, 1.44]
*3* m	15.8 ± 3.00	+9.19 (0.33)	8.54, 9.84	27.2	**<0.001**	+4.00 [3.71, 4.28]

Significant results are reported in **bold** characters. **Abbreviations**. CI, confidence interval; d, standardized mean difference; HARS, Hamilton Anxiety Rating Scale; HDRS, Hamilton Depression Rating Scale; LMM, Linear Mixed Model; M, Mean; *p*, statistical significance; PDQ-D5, Perceived Deficits Questionnaire for Depression–5 items; PSQI, Pittsburgh Sleep Quality Index; SD, Standard Deviation; SE, Standard Error; SF-36, Short Form Health Survey Questionnaire (PCS: Physical Component Score); WHO-5, World Health Organization-Five Well-Being Index. *Note*: Means are observed values; change estimates, *t-* and *p*-values are from linear mixed models adjusted for age, sex, illness duration, and concomitant medication.

## Data Availability

Authors do not have permission to share the data.

## Supplementary Materials

The following supporting information can be downloaded at: https://www.mdpi.com/article/10.3390/jcm15176874/s1, Supplementary Table S1: Diagnostics of the linear mixed models; Supplementary Table S2: Item-level changes in the HDRS and HARS physical items (n = 62); Supplementary Table S3: Comparison of the primary analysis and the deterministic boundary scenario for all outcomes; Supplementary Table S4: Exploratory time × concomitant psychotropic medication interaction models for all study outcomes; Supplementary Table S5: Estimated within-subgroup change from baseline, by concomitant psychotropic medication status.
