# Supplementary material for: Extended-Release Trazodone in Comorbid Major Depressive and Sedative–Hypnotic/Anxiolytic Use Disorders: A Real-World Preliminary Evaluation of Multidimensional Outcomes
Source: J Clin Med. 2026 Sep 4;15(17):6874. doi: 10.3390/jcm15176874 (PMC13566321; doi:10.3390/jcm15176874)
Supplement: Supplementary file 1 [file jcm-15-06874-s001.zip › jcm-4467382-supplementary.pdf]

Supplementary Material

Supplementary Table S1. Diagnostics of the linear mixed models.

Outcome	Shapiro–Wilk W	p	Residual Skew	Residual variance across fitted values	Random Intercepts
**HDRS_Total**	0.975	0.008	Mild, positive	Homogeneous	Symmetrical
**HDRS_Physical**	0.980	0.029	Mild, positive	Homogeneous; narrowing near scale floor	Symmetrical
**HARS_Total**	0.983	0.063	Mild, positive	Homogeneous	Symmetrical
**HARS_Physical**	0.989	0.313	Not appreciable	Homogeneous	Symmetrical
**SF-36 PCS**	0.993	0.692	Not appreciable	Homogeneous	Symmetrical
**PDQ-D5**	0.991	0.421	Not appreciable	Homogeneous	Symmetrical
**PSQI**	0.982	0.049	Mild, positive	Homogeneous; narrowing near scale floor	Symmetrical
**WHO-5**	0.982	0.044	Mild, negative	Homogeneous	Symmetrical

Shapiro–Wilk test computed on the conditional residuals (a non-significant result indicates that no departure from normality was detected). Skew, homogeneity of residual variance and symmetry of the random intercepts were assessed by visual inspection of Q–Q plots and histograms of the conditional residuals, residual-versus-fitted plots, and histograms of the estimated random intercepts. Since in the WHO-5 higher scores indicate better outcome, the direction of the skew is reversed with respect to the symptom measures.

Supplementary Table S2. Item-level changes in the HDRS and HARS physical items (n = 62).

Item	Baseline (M ± SD)	3 months (M ± SD)	W	p	p (BH)	r
**HDRS**						
4. Early insomnia	1.03 ± 0.70	0.47 ± 0.54	483.0	<0.001	<0.002	0.95
5. Middle insomnia	0.77 ± 0.64	0.23 ± 0.42	545.5	<0.001	<0.002	0.95
6. Late insomnia	0.47 ± 0.56	0.18 ± 0.43	180.5	<0.001	<0.002	0.90
11. Somatic anxiety	1.13 ± 0.64	0.31 ± 0.56	929.0	<0.001	<0.002	0.96
12. Gastrointestinal	0.90 ± 0.65	0.13 ± 0.34	780.0	<0.001	<0.002	1.00
13. General somatic	0.74 ± 0.63	0.15 ± 0.47	595.0	<0.001	<0.002	0.89
14. Genital	0.53 ± 0.50	0.11 ± 0.37	406.0	<0.001	<0.002	0.87
16. Loss of weight	0.55 ± 0.59	0.18 ± 0.43	300.5	<0.001	<0.002	0.85
**HARS**						
7. Muscular	1.15 ± 0.77	0.79 ± 0.68	226.0	<0.001	<0.002	0.79
8. Sensory	1.19 ± 0.81	0.71 ± 0.61	393.0	<0.001	<0.002	0.81
9. Cardiovascular	1.23 ± 0.84	0.50 ± 0.54	595.0	<0.001	<0.002	1.00
10. Respiratory	1.32 ± 0.81	0.50 ± 0.50	630.0	<0.001	<0.002	1.00

Item	Baseline (M ± SD)	3 months (M ± SD)	W	p	p (BH)	r
11. Gastrointestinal	1.15 ± 0.77	0.40 ± 0.50	741.0	<0.001	<0.002	1.00
12. Genitourinary	1.00 ± 0.75	0.44 ± 0.50	465.0	<0.001	<0.002	1.00
13. Autonomic	0.73 ± 0.81	0.48 ± 0.57	66.0	0.003	0.003	1.00

Note: Wilcoxon signed-rank tests comparing baseline and three months in the 62 patients completing both assessments. W, Wilcoxon test statistic; r, rank-biserial correlation; BH, Benjamini–Hochberg correction applied across the items. This analysis was not planned a priori and is reported as exploratory.

Supplementary Table S3. Comparison of the primary analysis and the deterministic boundary scenario for all outcomes.

	Primary analysis	Boundary scenario	
	F (time)	*F* (time)	Endpoint EMM (primary → sensitivity)
**HDRS – Total**	344.2	192.9	6.44 → 7.69
**HDRS – Physical**	200.7	138.8	2.08 → 2.61
**HARS – Total**	258.0	160.6	10.9 → 12.2
**HARS – Physical**	120.9	95.4	3.81 → 4.31
**SF-36 PCS**	243.5	123.6	49.8 → 47.6
**PDQ-D5**	179.5	112.7	4.82 → 5.73
**PSQI**	158.5	100.9	5.14 → 6.32

	Primary analysis	Boundary scenario	
**WHO-5**	384.3	199.7	15.73 → 14.52

All time effects were significant at p < 0.001 in both the primary and sensitivity analyses.

Abbreviations. EMM, estimated marginal mean; *F*, F-statistic for the omnibus effect of time; HARS, Hamilton Anxiety Rating Scale; HDRS, Hamilton Depression Rating Scale; PDQ-D5, Perceived Deficits Questionnaire for Depression – 5 items; PSQI, Pittsburgh Sleep Quality Index; SF-36 PCS, 36-Item Short Form Survey – Physical Component Score; WHO-5, World Health Organization-Five Well-Being Index.

Note. The primary analysis retained all 74 participants with all available observations. This boundary scenario imputed missing three-month values under a conservative deterministic assumption: baseline-observation-carried-forward for patients who relapsed into SHA use (n = 5) and last-observation-carried-forward for the remaining discontinuers (n =7). For all outcomes, the effect of time remained significant, with estimates attenuated but directionally unchanged, indicating robustness of the primary findings to participant attrition. For the positively scored measures (SF-36 PCS and WHO-5), endpoint values shifted downward under imputation; for all other measures, scores shifted upward, in both cases reflecting reduced improvement.

Table S4. Exploratory time × concomitant psychotropic medication interaction models for all study outcomes.

Outcome	Time × medication interaction	p	Between-group difference in change, T0–T1, β (95% CI)	Between-group difference in change, T0–T3, β (95% CI)	Main effect of concomitant medication
HDRS, total score	F(2, 137) = 4.18	.018	−0.92 (−2.90 to 1.07)	2.09 (0.00 to 4.18)	F(1, 69) = 0.05, p = .823
HDRS, somatic subscale	F(2, 136) = 1.70	.188	−0.33 (−1.42 to 0.76)	0.73 (−0.42 to 1.87)	F(1, 69) = 0.94, p = .337
HARS, total score	F(2, 136) = 0.33	.722	0.84 (−1.38 to 3.06)	0.10 (−2.22 to 2.43)	F(1, 69) = 2.61, p = .113
HARS, somatic subscale	F(2, 135) = 0.04	.961	0.04 (−1.18 to 1.27)	−0.14 (−1.43 to 1.15)	F(1, 69) = 0.09, p = .767
PDQ-D-5, total score	F(2, 133) = 0.29	.750	−0.23 (−1.96 to 1.50)	0.46 (−1.36 to 2.28)	F(1, 69) = 0.20, p = .656
PSQI, global score	F(2, 134) = 0.98	.380	−0.84 (−3.04 to 1.35)	0.79 (−1.53 to 3.11)	F(1, 69) = 0.62, p = .433
SF-36, PCS	F(2, 133) = 1.30	.277	−1.76 (−4.65 to 1.13)	0.58 (−2.47 to 3.62)	F(1, 69) = 1.66, p = .204
WHO-5, total score	F(2, 132) = 0.04	.961	0.14 (−1.52 to 1.79)	0.25 (−1.50 to 1.99)	F(1, 69) = 1.58, p = .215

Note. Linear mixed-effects models with a random intercept for participant, fitted for each outcome and including the time × concomitant psychotropic medication interaction, the main effects of both terms, and age, sex and illness duration as covariates. Degrees of freedom were estimated with the Satterthwaite approximation. Number of observations = 210; number of participants = 74 (concomitant psychotropic medication: n = 53; trazodone monotherapy: n = 21). Between-group differences in change are the interaction contrasts, expressed as the additional change in the concomitant-medication subgroup relative to the monotherapy subgroup; negative values on HDRS, HARS, PDQ-D-5 and PSQI and positive values on SF-36 PCS and WHO-5 indicate greater improvement in the concomitant-medication subgroup. T0 = baseline; T1 = intermediate assessment (1 month); T3 = endpoint. Because these models include the interaction term, the marginal effect of time corresponds to the unweighted average of the two subgroup-specific effects and therefore differs slightly from the corresponding estimates of the primary models reported in Table 2, in which a single common trajectory is estimated across the whole sample. These analyses are exploratory and are reported without correction for multiplicity; across eight interaction tests the Bonferroni-corrected threshold is $p = .00625$, and the single nominally significant result (HDRS total score) does not meet it under either Bonferroni or Benjamini–Hochberg correction. Bonferroni-corrected pairwise comparisons between the two subgroups at each individual assessment point were non-significant for every outcome (all $p \geq .667$). Concomitant medication was modelled as a binary variable (any vs none); the sample did not allow separate modelling of individual medication classes. HDRS = Hamilton Depression Rating Scale; HARS = Hamilton Anxiety Rating Scale; PDQ-D-5 = Perceived Deficits Questionnaire-Depression, 5-item; PSQI = Pittsburgh Sleep Quality Index; SF-36 PCS = 36-Item Short Form Health Survey, Physical Component Summary; WHO-5 = World Health Organization-Five Well-Being Index.

Table S5. Estimated within-subgroup change from baseline, by concomitant psychotropic medication status.

**Outcome**	**Trazodone monotherapy, T0–T1, Δ (95% CI)**	**Trazodone monotherapy, T0–T3, Δ (95% CI)**	**Concomitant psychotropics, T0–T1, Δ (95% CI)**	**Concomitant psychotropics, T0–T3, Δ (95% CI)**
HDRS, total score	−4.27 (−5.95 to −2.58)	−12.55 (−14.31 to −10.78)	−5.18 (−6.24 to −4.12)	−10.46 (−11.59 to −9.32)
HDRS, somatic subscale	−1.93 (−2.86 to −1.01)	−5.03 (−6.00 to −4.06)	−2.26 (−2.84 to −1.68)	−4.30 (−4.92 to −3.68)
HARS, total score	−4.87 (−6.76 to −2.97)	−10.26 (−12.23 to −8.29)	−4.03 (−5.21 to −2.84)	−10.16 (−11.41 to −8.90)
HARS, somatic subscale	−2.20 (−3.24 to −1.16)	−3.72 (−4.81 to −2.63)	−2.16 (−2.81 to −1.50)	−3.86 (−4.56 to −3.16)
PDQ-D-5, total score	−2.40 (−3.87 to −0.93)	−6.96 (−8.49 to −5.42)	−2.63 (−3.56 to −1.71)	−6.50 (−7.48 to −5.51)
PSQI, global score	−1.87 (−3.73 to −0.001)	−8.51 (−10.47 to −6.56)	−2.71 (−3.88 to −1.54)	−7.73 (−8.98 to −6.47)
SF-36, PCS	4.86 (2.40 to 7.32)	12.49 (9.92 to 15.07)	3.10 (1.56 to 4.64)	13.07 (11.42 to 14.72)
WHO-5, total score	2.60 (1.19 to 4.01)	9.02 (7.54 to 10.49)	2.74 (1.85 to 3.62)	9.26 (8.32 to 10.21)

Note. Simple effects of time within each subgroup, estimated as model contrasts from the models reported in Table S4, with 95% Wald confidence intervals. Negative values on HDRS, HARS, PDQ-D-5 and PSQI and positive values on SF-36 PCS and WHO-5 indicate clinical improvement. The effect of time was significant within both subgroups for every outcome (all $p \leq .050$); all contrasts were significant at $p < .001$ except the T0–T1 contrast in the monotherapy subgroup on the PDQ-D-5 ($p = .002$) and the PSQI ($p = .050$). Within-subgroup simple effects are reported without correction for multiplicity. These estimates are exploratory; the monotherapy subgroup is small and the two subgroups are unbalanced, so the absence of interactions surviving correction for multiplicity in Table S4 should not be interpreted as positive evidence of no effect modification. Abbreviations as in Table S4.
